# TLR4 interaction with PIEZO1 facilitates the 5-HT-mediated intestinal motility dysfunction in offspring mice induced by LPS exposure during pregnancy

**DOI:** 10.1016/j.gendis.2025.101707

**Published:** 2025-06-05

**Authors:** Ruifang Luo, Yuan Miao, Riqiang Hu, Fang Lin, Junyan Yan, Ting Yang, Lu Xiao, Zhujun Sun, Yuting Wang, Jie Chen

**Affiliations:** a Growth, Development and Mental Health Center of Children and Adolescents, Children’s Hospital of Chongqing Medical University, Chongqing Key Laboratory of Child Neurodevelopment and Cognitive Disorders, National Clinical Research Center for Child Health and Disorders, Ministry of Education Key Laboratory of Child Development and Disorders, Chongqing 404100, China; b Digestive Department, Children’s Hospital of Chongqing Medical University, Chongqing 404100, China

**Keywords:** 5-HT, Enterochromaffin cell, Intestinal motility, Prenatal LPS exposure, TLR4

## Abstract

Several factors during pregnancy, such as changes in serotonin (5-HT) levels, can affect intestinal function in offspring mice. The role of 5-HT in regulating intestinal motility after lipopolysaccharide (LPS) exposure during pregnancy is unclear. In this study, *Tlr4*^fl/fl^ and *Tlr4*^▵IEC^ mice were injected with LPS or phosphate-buffered saline during pregnancy to obtain prenatal LPS-exposed or non-exposed offspring mice. Changes in intestinal morphology, motility, and the TLR4 and 5-HT signaling pathways were examined in male offspring mice. The role of TLR4 in regulating 5-HT secretion was investigated in the BON-1 enterochromaffin cell line. In the prenatal LPS-exposed *Tlr4*^fl/fl^ group, offspring mice exhibited colonic mucosal injury and faster intestinal motility, but these effects were absent when TLR4 was knocked out in intestinal epithelial cells. The TLR4 and 5-HT signaling pathways were activated in the colon of prenatal LPS-exposed *Tlr4*^fl/fl^ offspring mice but were inactivated in prenatal LPS-exposed *Tlr4* knockout offspring mice. In BON-1 cells, TLR4 interacted with the calcium ion channel PIEZO1, causing calcium influx and promoting 5-HT secretion. This process was disrupted by the TLR4 inhibitor TAK242. LPS exposure during pregnancy affected intestinal motility in offspring mice by activating TLR4 pathways in the colon and increasing 5-HT secretion from enterochromaffin cells. The effects of LPS on the intestine might be explained by the interaction between TLR4 and PIEZO1, suggesting that TLR4 is related to abnormal intestinal motility in offspring mice exposed to LPS during pregnancy.

## Introduction

Human health outcomes are shaped by events occurring during pregnancy and early childhood,^1^ and infection during pregnancy is a significant risk factor for health problems in offspring.^2^, ^3^, ^4^ Several studies have revealed that maternal infection during pregnancy can compromise the integrity of the intestinal barrier and increase the risk of intestinal inflammatory diseases in offspring.^5^, ^6^, ^7^, ^8^ Another study has found that maternal prenatal exposure to lipopolysaccharide (LPS) results in intestinal barrier damage in offspring, which persists into adulthood. However, despite the considerable focus on the intestinal barrier function of prenatally infected offspring, research on their intestinal motility remains limited.

Intestinal motility plays a key role in maintaining normal intestinal physiological function.^10^ It is regulated by various intestinal neurotransmitters secreted by enteroendocrine cells, among which serotonin (5-HT) is of particular interest to intestinal function.^11^ The neurotransmitter 5-HT is mainly secreted by enterochromaffin (EC) cells, and its extracellular levels are regulated by the sodium-dependent serotonin transporter solute carrier family 6 member 4 (SLC6A4).^12^ EC cells are neurosensory cells located in the intestinal epithelium that convert the perceived intestinal environmental signals into 5-HT secretion, thereby contributing to intestinal motility.^13^ Various factors influence 5-HT secretion in the gut, including nutrients, microbial communities, and host-derived signaling hormones.^14^, ^15^, ^16^ A recent study found that EC cells detect alterations in the intestinal inflammatory environment and release 5-HT to enhance intestinal peristalsis, thus facilitating the expulsion of *Trichuris muris*.^17^ Another study reported that the gut microbiota or nutritional metabolites can stimulate EC surface receptors to produce and secrete 5-HT, thereby improving intestinal motility.^18^ The neurotransmitter 5-HT regulates intestinal peristalsis by binding to its receptors, of which 5-HT2, 5-HT3, and 5-HT4 have been studied and implicated in intestinal peristalsis.^19^, ^20^, ^21^ Our previous studies found that prenatal LPS-exposed offspring rats had higher levels of 5-HT in the colon.^22^ However, more research is needed to understand its role in intestinal motility and how it interacts with specific regulatory factors.

By maintaining the integrity of the gut barrier, toll-like receptor 4 (TLR4) contributes to the homeostasis of the gut environment.^23^ Recent studies have indicated that TLR4 can influence the 5-HT-induced contractile response by altering the expression pattern of 5-HT receptors, and knocking out *Tlr4* in mice leads to the attenuation of 5-HT-induced colon contraction.^24^ According to a previous study, TLR4 activation regulates the expression of nucleotide binding oligomerization domain containing 1 (NOD1), which inhibits SLC6A4 activity through the extracellular signal-regulated kinase (ERK) signaling pathway.^25^ However, it has also been found that 5-HT increases the expression of TLR4 and *E. coli* colonization in the gut of inflammatory bowel disease.^26^ Therefore, the regulatory interplay between TLR4 and 5-HT remains controversial. Despite the observed correlation between the two, the precise regulatory mechanisms require further investigation.

This study aims to investigate the key role of TLR4 in regulating the 5-HT signaling pathway in the intestines of offspring mice to understand the mechanism by which 5-HT supports intestinal motility. Initially, intestinal epithelial *Tlr4* conditional knockout (*Tlr4*^ΔIEC^) mice were constructed and injected with LPS during gestation to examine alterations in intestinal dynamics in the resulting offspring. Subsequently, a correlation between intestinal TLR4 signaling and 5-HT signaling was found through RNA sequencing. Ultimately, *in vitro* experiments showed that TLR4 interacted with Piezo-type mechanosensitive ion channel component 1 (PIEZO1), increasing Ca^2+^ influx and promoting 5-HT secretion.

## Material and methods

### Animals

**Figure 1:**
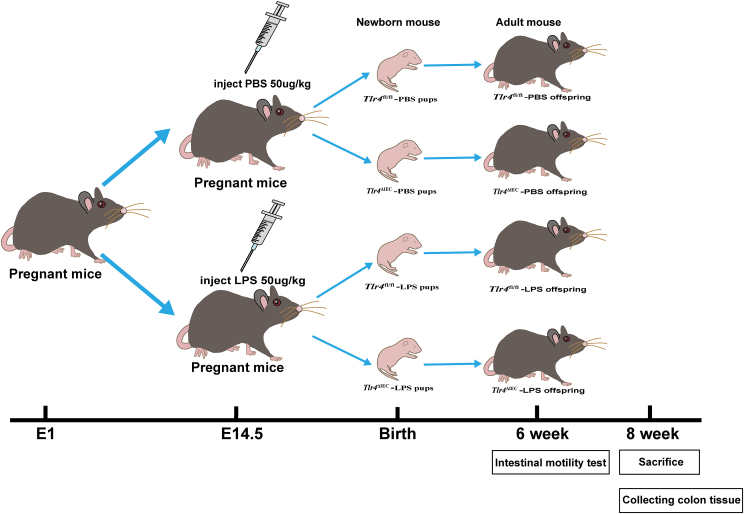
Schematic diagram of the four groups. *Tlr4*^fl/fl^ mice were crossbred with *Tlr4*^ΔIEC^ mice, and the female mice were intraperitoneally injected with 50 μg/kg of lipopolysaccharide (LPS) or an equivalent volume of phosphate buffer saline (PBS) at gestational day 14.5. After genotyping, the offspring mice were divided into four groups: *Tlr4*^fl/fl^-PBS, *Tlr4*^fl/fl^-LPS, *Tlr4*^ΔIEC^-PBS, and *Tlr4*^ΔIEC^-LPS. Intestinal motility and fecal water content were measured at 6 weeks of age. Colon tissue was collected at 8 weeks of age.

*Tlr4*-floxed (*Tlr4*^fl/fl^) and Villin-Cre mice were obtained from the Cyagen Transgenic Animal Center (Suzhou, China). Embryonic intestine-epithelium-specific *Tlr4* knockout (*Tlr4*^△IEC^) mice were generated by crossing Villin-Cre mice with *Tlr*4^fl/fl^ mice. This study was approved by the Animal Ethics Committee of the Children’s Hospital of Chongqing Medical University (Chongqing, China) (IACUC Issue No: CHCMU-IACUC2022062914). Eight-week-old *Tlr4*^△IEC^ and *Tlr4*^fl/fl^ mice were mated overnight. Then, females were examined for vaginal plugs the following morning. Female mice were randomly injected intraperitoneally with LPS (MilliporeSigma, Burlington, Massachusetts, USA) at 50 μg/kg or an equivalent volume of phosphate buffer saline (PBS) at 14.5 days of gestation, as shown in Figure 1. Since hormone levels at different periods quickly affect intestinal development in female mice, only male offspring mice were used in this study.^27^ DNA was extracted from the tail tissue of 3-week-old offspring mice for genotyping, and then offspring mice were divided into four groups: *Tlr4*^fl/fl^-PBS, *Tlr4*^fl/fl^-LPS, *Tlr4*^△IEC^-PBS, and *Tlr4*^△IEC^-LPS. The intestinal motility test was performed on 6-week-old male mice. At eight weeks, the mice were euthanized with phenobarbital sodium. The entire middle part of the colon tissue of each mouse was collected on ice, snap-frozen in liquid nitrogen, and stored in a −80 °C freezer for subsequent experiments.

### Hematoxylin and eosin staining

The colon tissue sample was embedded in paraffin and cut into sections at 5 μm thickness using a Leica RM2265 paraffin microtome (Leica Microsystems GmbH, Wetzlar, Germany). The sections were deparaffinized, hydrated, and stained with hematoxylin and eosin according to standard procedures, sealed and dried, and scanned by an SQS-40P slide scanning system (Shengqiang Technology Co., Ltd., Shenzhen, China). Digital pathological sections were analyzed using the ImageJ Pro Plus 6.0 software (National Institutes of Health, Bethesda, Maryland, USA).

### Gut motility assessments

After fasting overnight, we administered to offspring mice 300 μL of a carmine red (MilliporeSigma) solution by gavage, and their stool color was observed every 10 min. The time from gavage to discharge of carmine red was recorded as the gastrointestinal transit time. We measured colonic transit time using 3 mm-diameter glass beads pushed approximately 3 cm from the anus to the colon of the mouse, and the time of glass bead expulsion was recorded as the colonic transit time. The water content of feces was measured after overnight fasting by collecting, weighing, and recording the weight of the feces of offspring mice as W1. Then, after baking the collected feces in an oven at 60 °C for 24 h, the weight of the dried feces was determined and recorded as W2. Fecal water content was calculated using the formula: fecal water content = (W1 − W2)/W1.

### RNA sequencing analysis

The entire middle part of the colon tissue of the mice from each of the four groups was subjected to RNA sequencing analysis. After extracting total RNA from each sample, RNA concentration and purity were determined using a NanoDrop 2000 spectrometer (Thermo Fisher Scientific Inc., Waltham, Massachusetts, USA). Subsequently, after enriching the mRNA using Oligo (dT) beads, the mRNA was fragmented by adding lysis buffer, and small fragments of approximately 300 bp were isolated using magnetic beads. The mRNA was used as a template to synthesize the first strand of cDNA using reverse transcriptase, followed by the synthesis of the second strand of cDNA using DNA polymerase. Then, the End Repair Mix and poly(A) were added to the synthesized cDNA fragment. Ultimately, mRNA sequencing was performed by Majorbio Biotech Co., Ltd. (Shanghai, China) using the Illumina Novaseq 6000 sequencing platform (Illumina Inc.). DESeq2 was used to identify differentially expressed genes (DEGs) with fold change >2 and *P*-value <0.05. The function of DEGs was investigated by performing Kyoto Encyclopedia of Genes and Genomes (KEGG) pathway enrichment analyses. We then focused on the most relevant primary pathway linked to intestinal peristaltic function in the enriched KEGG pathway, which was the 5-HT pathway. We then constructed gene sets for the 5-HT and TLR4 pathways, and the expression of the two gene sets in the four sample groups was analyzed. Finally, we looked at related studies and websites to find genes that are closely related to our research and checked their expression levels using quantitative PCR.

### Western blotting analysis

We extracted the total protein from the middle part of the colon tissue of the mice from each of the four groups using the total protein extraction kit (KeyGen Biotech, Nanjing, China), according to the manufacturer’s instructions. After separating the proteins by sodium dodecyl sulfate-polyacrylamide gel electrophoresis (SDS-PAGE), the proteins were electro-transferred onto 0.22 μm polyvinylidene difluoride (PVDF) membranes, which were then blocked with blocking buffer (5% non-fat dry milk and 0.05% Tween-20 in phosphate-buffered saline) at 4 °C. Subsequently, the membranes were individually incubated with the respective primary antibodies at 4 °C overnight. The primary antibodies used included anti-TLR4 (Cat#SC-293072; Santa Cruz Biotechnology Inc., Santa Cruz, California, USA), anti-RELA (Cat#8242s; Cell Signaling Technology Inc., Danvers, Massachusetts, USA), anti-phosphoRELA (Cat#3033s; Cell Signaling Technology Inc.), anti-CHUK (Cat#61294s; Cell Signaling Technology Inc), anti-PTGS2 (Cat#RT1159; Huabio, Hangzhou, China), anti-TPH1 (Cat#CY5574; Abways, Shanghai, China), anti-SLC6A4 (Cat#382321; Zen-Bioscience, Chengdu, China), anti-HTR2C (Cat#381979; Zen-Bioscience), anti-HTR3A (Cat#120185; Zen-Bioscience), anti-HTR4 (Cat#860013; Zen-Bioscience), and anti-ACTB (Cat#AC026; ABclonal Biotech Co., Ltd., Wuhan, China). Following incubation with the primary antibodies, the membranes were washed and incubated with the appropriate horseradish peroxidase (HRP)-conjugated secondary antibodies (ATGen Co., Ltd., Solana Beach, California, USA) at room temperature for 1 h. After detection of the immunoreactive proteins, the membranes were washed with an antibody-stripping buffer (KeyGen Biotech). Subsequently, the membranes were incubated with anti-ACTB primary antibody at room temperature for 2 h, followed by incubation with the appropriate secondary antibody to detect the immunoreactive ACTB protein band. The target and internal control proteins are shown on intact membranes without clipping. The immunoreactive protein bands on the membranes were visualized using a chemiluminescent HRP substrate (MilliporeSigma). The images were captured using a Syngene GBox Imaging System (Syngene, Cambridge, UK).

### RNA isolation and quantitative real-time PCR

Total RNA was extracted using Trizol reagent (Beijing Solarbio Science & Technology Co., Ltd., Beijing, China). The mRNA was reverse-transcribed using a high-capacity cDNA reverse transcription kit (Takara Bio, Otsu, Shiga, Japan) and subjected to quantitative real-time PCR analysis using SYBR Green I Master Mix reagent (QIAGEN GmbH, Hilden, Germany) on a Bio-Rad CFX system (Bio-Rad Laboratories, Hercules, California, USA). The sequences of the primers synthesized and used in this study are listed in Table S1.

### Enzyme-linked immunosorbent assay (ELISA)

The concentration of 5-HT in colon tissue, fecal samples, and cell supernatants was determined with ELISA under the manufacturer’s protocol (Ruixin Biotech Co., Ltd., Quanzhou, China).

### Cell culture

The BON-1 cell line was obtained from the Cancer Institute, Hospital of the Chinese Academy of Medical Sciences (Beijing, China). Cells were maintained in RPMI 1640 medium supplemented with 5% fetal bovine serum in a humidified incubator at 37 °C with 5% CO_2_. Before the LPS treatment, the cells were treated with the TLR4 inhibitor TAK-242 (M4838; ABMole BioScience Inc., Houston, Texas, USA) at 10 μmol/L for 1 h. Cells were then harvested after 12 h of treatment with LPS or PBS. The four cell treatment groups were as follows: PBS, PBS + TAK-242, LPS, and LPS + TAK-242.

### Immunofluorescence staining

The cells were fixed with 4% paraformaldehyde at room temperature for 20 min, and then permeated with 0.2% Triton X-100 (Beyotime Biotechnology, Shanghai, China) for 10 min. After rinsing with PBS, cells were blocked with 5% bovine serum albumin (FA016; GenView Corp., Houston, Texas, USA) at room temperature for 30 min. The corresponding primary antibodies, including anti-TLR4 (Cat#19811-1-Ap; Proteintech Group Inc., Rosemont, Illinois, USA), anti-5-HT (Cat#5-HT-H209; GeneTex Inc., Irvine, California, USA), and anti-PIEZO1 (Cat#28511-1-AP; Proteintech Group Inc.), were incubated at 4 °C overnight. The cell nuclei were stained by incubating with the fluorescent dye 4′,6-diamidino-2-phenylindole (Abcam, Cambridge, UK) at room temperature in the dark for 45 min. Then, the cells were incubated with the appropriate fluorescent secondary antibody (Abcam) at room temperature in the dark for 1 h. The tissue section staining procedure was the same as above. After sealing, the cell slides and tissue sections were observed by confocal fluorescence microscopy using a Nikon Eclipse 80i confocal fluorescence microscope (Nikon Instruments, Tokyo, Japan).

### Calcium imaging

The BON-1 cells were seeded in 35-mm confocal dishes for calcium imaging of each treatment group. When the cells reached 70%–80% confluency, intracellular calcium levels were determined using the Fluo-4 Calcium Assay Kit (Cat#S1061S, Beyotime Biotechnology), which is based on the detection of the fluorescent light emitted by the calcium indicator Fluo-4 AM upon binding to Ca^2+^. All procedures were performed in strict accordance with the manufacturer’s instructions. The Ca^2+^ influx was imaged under a fluorescence microscope, and the fluorescence intensity of Ca^2+^ was analyzed using the Nikon analysis software.

### Co-immunoprecipitation

Cells were collected and lysed with radioimmunoprecipitation assay (RIPA) buffer (KeyGen Biotech) to obtain proteins, which were then co-immunoprecipitated with the TLR4 antibody using a co-immunoprecipitation kit (Beyotime Biotechnology). Briefly, the TLR4 antibody was bound to proteins to form immune complexes, which were then immunoprecipitated with magnetic beads and pre-washed to remove non-specifically bound proteins. TLR4 and PIEZO1 were detected in the immune complexes by SDS-PAGE on a polyacrylamide gel (Epizyme Biomedical Technology, Shanghai, China), using the same procedure as for western blotting analysis.

### Statistical analysis

Differences among the different groups were evaluated using a two-way analysis of variance (ANOVA), followed by a Bonferroni post hoc test. When statistically significant interactions were detected, the Bonferroni post hoc test was used to determine significant differences among the experimental groups. A *t*-test was used to analyze the effect of LPS and TLR4 when no statistically significant interaction was found between LPS and TLR4. Genotyping and quantitative PCR data from drug-treated cells were analyzed using a *t*-test and one-way ANOVA, respectively. Statistical analyses were performed using GraphPad Prism 8.0 (GraphPad Software Inc., San Diego, California, USA). Data were expressed as mean ± standard error of the mean. Statistical significance was defined as ∗*P* < 0.05, ^∗∗^*P* < 0.01, ^∗∗∗^*P* < 0.001, and ^∗∗∗∗^*P* < 0.0001.

## Results

### Prenatal LPS treatment increased intestinal mucosal damage and gastrointestinal motility in *Tlr4*^fl/fl^ offspring mice but not in *Tlr4*^ΔIEC^ mice

**Figure 2:**
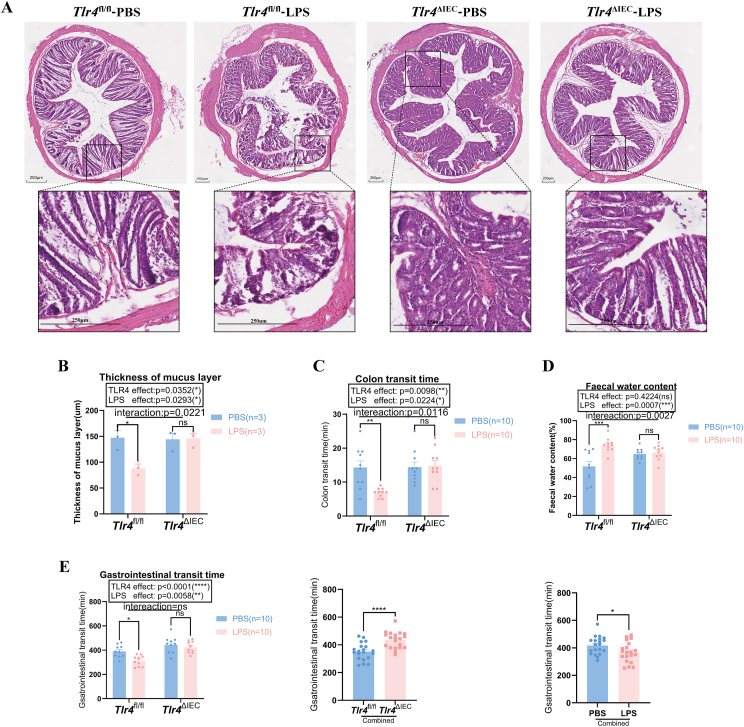
Morphological observation and intestinal motility changes in the colon of offspring mice. **(A)** The hematoxylin and eosin staining of colon samples from the offspring of *Tlr4*^fl/fl^ and *Tlr4*^ΔIEC^ mice treated with phosphate buffer saline (PBS) or lipopolysaccharide (LPS) provided an overview of the colon in both groups. Scale bar = 250 μm (*n* = 3 per group). **(B)** Histogram of colonic tissue mucosal layer thickness quantification in offspring mice of the four groups. **(C)** The colonic transit time in the offspring of *Tlr4*^fl/fl^ and *Tlr4*^ΔIEC^ mice after treatment with LPS or PBS (*n* = 10 per group; two-way ANOVA). **(D)** Fecal water content in the *Tlr4*^fl/fl^ and *Tlr4*^ΔIEC^ offspring with or without LPS exposure (*n* = 10 per group; two-way ANOVA). **(E)** The gastrointestinal transit time of the offspring mice in the *Tlr4*^fl/fl^ and *Tlr4*^ΔIEC^ groups with or without LPS treatments (*n* = 10 per group; two-way ANOVA). In the combined *Tlr4*^fl/fl^ and *Tlr4*^ΔIEC^ groups, as well as in the combined PBS and LPS groups (*n* = 20 per group), *Tlr4* knockout or LPS treatment independently affected gastrointestinal transit time. Data were presented as mean ± standard error of the mean. The term “Interaction” denotes the effect of LPS on the *Tlr4*^ΔIEC^ mice compared with *Tlr4*^fl/fl^ mice. ns, not significant; ∗*P* < 0.05, ∗∗*P* < 0.01, ∗∗∗∗*P* < 0.0001, and ∗∗∗∗*P* < 0.0001.

**Figure S1:**
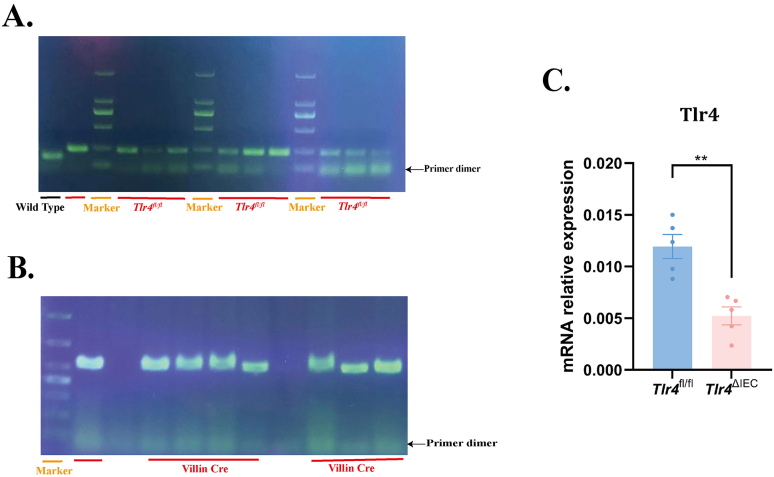
Genotype identification and quantitative PCR gene expression analysis. **(A)** Agarose gel image of the PCR amplified products using Flox primers observed by a gel imaging system. **(B)** Agarose gel image of the PCR amplified products using Villin-Cre primer observed by a gel imaging system. **(C)***Tlr4* mRNA expression levels in *Tlr4*^fl/fl^ and *Tlr4*^ΔIEC^ mice detected by quantitative PCR analysis (*n* = 5 per group; student’s *t*-test). Data were expressed as mean ± standard error of the mean; ns, not significant; ∗∗*P* < 0.01. TLR4, toll-like receptor 4.

Before the corresponding experiments, each mouse was genotyped, and quantitative PCR analysis was performed to investigate the effect of intestinal *Tlr4* knockout on gene expression, and typical experimental results are shown in Figure S1. The offspring of LPS-exposed *Tlr4*^fl/fl^ mice showed a reduction in the thickness of the colonic mucus layer and compromised integrity of the colonic mucus layer compared with PBS-treated mice (*P* = 0.0197) (Fig. 2A, B). No significant changes were observed in the submucosa, muscularis, and serous layers between the two groups. In contrast, *Tlr4*^ΔIEC^ mice showed no significant differences in colon structure between LPS and PBS treatment groups (Fig. 2A). Additionally, as shown in Figure 2C, the colonic transit time in *Tlr4*^fl/fl^ offspring mice of the LPS group was shorter than that in those of the PBS group (*P* = 0.0063), but there was no significant difference in *Tlr4*^ΔIEC^ offspring mice. Similarly, the fecal water content in *Tlr4*^fl/fl^ offspring mice of the LPS group was higher than that in those of the PBS group (*P* = 0.0001), but there was no significant difference in *Tlr4*^ΔIEC^ offspring mice between the two groups (Fig. 2D). The interaction between the LPS treatment and the *Tlr4*-tissue specific knockout also had a significant effect on colonic transit time and fecal water content, according to a Bonferroni post hoc analysis (*P* = 0.00116 and *P* = 0.0027, respectively). Prenatal LPS exposure significantly shortened the gastrointestinal transit time in *Tlr4*^fl/fl^ offspring mice but not in *Tlr4*^ΔIEC^ offspring mice (*P* = 0.0149), and there was no interaction effect between LPS treatment and the *Tlr4* knockout (Fig. 2E). After combining the two PBS and LPS groups, the gastrointestinal transit time was significantly increased in the combined *Tlr4*^ΔIEC^ group compared with the combined *Tlr4*^fl/fl^ group (*P* < 0.0001). Additionally, the gastrointestinal transit time in the combined LPS group was also statistically significantly lower than that in the combined PBS group following together with the two *Tlr4*^fl/fl^ and *Tlr4*^ΔIEC^ groups (*P* = 0.0280) (Fig. 2E).

These results suggest that prenatal LPS exposure impaired intestinal structure and increased fecal water content and gastrointestinal motility in *Tlr4*^fl/fl^ offspring mice, but had no significant effect on *Tlr4*^ΔIEC^ offspring mice.

### Prenatal LPS exposure activated the TLR4 signaling pathway in the intestine of *Tlr4*^fl/fl^ offspring mice but did not affect *Tlr4*^ΔIEC^ offspring mice

**Figure 3:**
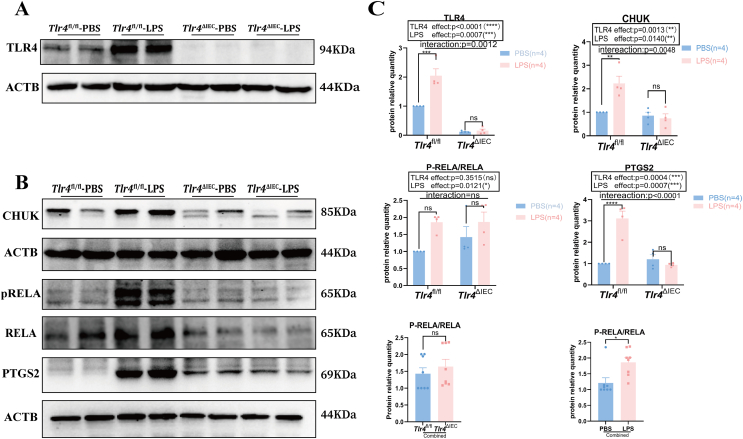
The levels of colonic TLR4 and related protein expression in *Tlr4*^fl/fl^ and *Tlr4*^ΔIEC^ offspring mice with or without prenatal LPS exposure. **(A)** The levels of colonic TLR4 protein expression in offspring mice (*n* = 4 per group). **(B)** The levels of colonic CHUK, RELA, P-RELA, and PTGS2 protein expression in the four groups (*n* = 4 per group). **(C)** The TLR4, CHUK, and PTGS2 protein expression levels in the four groups were normalized to ACTB, and the P-RELA to RELA ratio was used to evaluate the proportion of P-RELA quantitatively (*n* = 4 per group; two-way ANOVA). Data were presented as mean ± standard error of the mean. The term “Interaction” denotes the effect of LPS in the *Tlr4*^ΔIEC^ mice compared with *Tlr4*^fl/fl^ mice. ns, not significant; ∗*P* < 0.05, ∗∗*P* < 0.01, ∗∗∗*P* < 0.001, and ∗∗∗∗*P* < 0.0001. TLR4, toll-like receptor 4; LPS, lipopolysaccharide; CHUK, component of inhibitor of nuclear factor Kappa B kinase complex; PTGS2, prostaglandin-endoperoxide synthase 2; ACTB, actin beta.

The expression of proteins related to the TLR4 signaling pathway in the colon of 8-week-old offspring mice was analyzed to determine whether TLR4 played a role in LPS-induced maternal immune activation. The expression level of TLR4 protein was significantly increased in *Tlr4*^fl/fl^ mice of the LPS group compared with that in *Tlr4*^fl/fl^ mice of the PBS group (*P* = 0.0003). TLR4 protein expression was significantly reduced after conditional knockout of *Tlr4*, and LPS exposure could not induce *Tlr4* expression (Fig. 3A, C). Proteins downstream of TLR4 were also examined. The component of inhibitor of nuclear factor Kappa B kinase complex (CHUK, also known as inhibitor of nuclear factor kappa-B kinase subunit alpha (IKKA)) is an inhibitor kinase of RELA (RELA proto-oncogene, NF-κB subunit).^28^ Our results showed that the protein expression level of CHUK was higher in the colon of *Tlr4*^fl/fl^ mice of the LPS group than that in those of the PBS group (*P* = 0.0046), but its protein expression was not induced in *Tlr4*^ΔIEC^ mice of the LPS group. Additionally, there was an effect of the interaction between LPS exposure and *Tlr4* knockout in intestinal epithelium on both the TLR4 and CHUK protein levels (*P* = 0.0012 and *P* = 0.0048, respectively) (Fig. 3B, C). RELA, also known as P65, is a crucial functional subunit of the NF-kB complex. The ratio of phosphorylated-RELA (P-RELA) to total RELA is generally used to gain insight into the activation state of the NF-κB signaling pathway.^29^ We found that the P-RELA to RELA ratio showed an increasing trend with LPS exposure, regardless of whether it was in the colon of the offspring of the *Tlr4*^fl/fl^ or *Tlr4*^ΔIEC^ mice, and there was no significant interaction between LPS exposure and *Tlr4* knockout (Fig. 3B, C). After combining the two LPS groups with *Tlr4*^fl/fl^ and *Tlr4*^ΔIEC^ mice, the P-RELA to RELA ratio in the combined LPS group showed a significant increase compared with that in the combined PBS group (*P* = 0.0101). However, there was no significant difference between the combined LPS groups with *Tlr4*^fl/fl^ and *Tlr4*^ΔIEC^ mice. These results suggest that the P-RELA to RELA ratio in the colon of offspring mice was mainly affected by prenatal LPS exposure (Fig. 3B, C). Prostaglandin-endoperoxide synthase 2 (PTGS2, also known as COX-2) is regulated by TLR4 signaling and plays a vital role in proliferation and apoptosis in the intestine.^30^ The intestinal protein expression level of PTGS2 in *Tlr4*^fl/fl^ mice of the LPS group was significantly higher than that in *Tlr4*^fl/fl^ mice of the PBS group (*P* < 0.0001), but no difference was found between the LPS and PBS exposure after knocking out *Tlr4* in the intestinal epithelium. The Bonferroni post hoc analysis showed an interaction between LPS exposure and *Tlr4* knockout (*P* < 0.0001) (Fig. 3B, C).

These findings suggest that prenatal LPS exposure induced intestinal TLR4 signaling activation in *Tlr4*^fl/fl^ offspring but not in *Tlr4*^ΔIEC^ mice.

### The 5-HT signaling pathways were involved in intestinal transit dysfunction in prenatal LPS-exposed offspring mice

**Figure 4:**
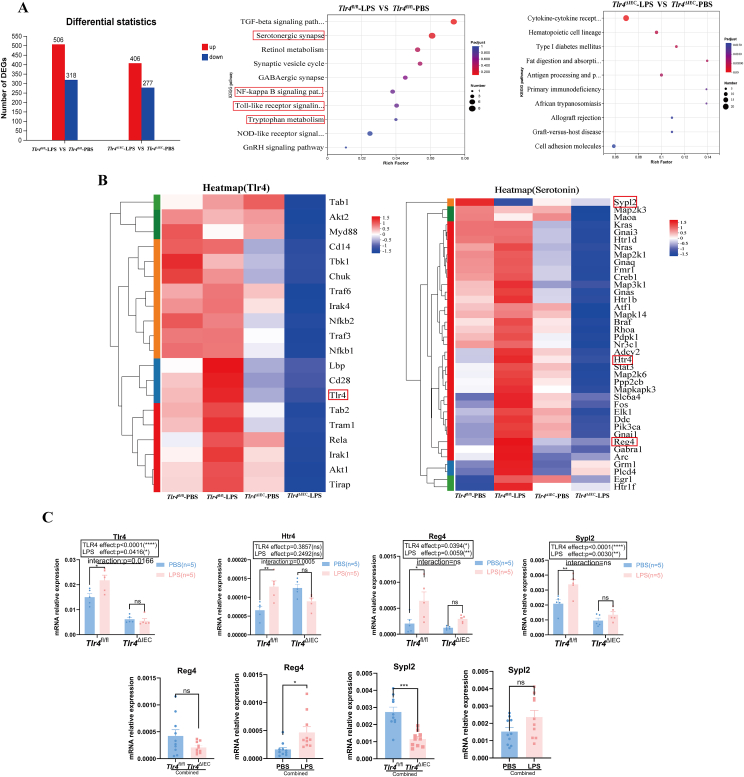
Gene expression profile in both the *Tlr4*^fl/fl^ group and *Tlr4*^ΔIEC^ group with or without prenatal LPS exposure. **(A)** Analysis of differentially expressed genes (DEGs) and Kyoto Encyclopedia of Genes and Genomes (KEGG) pathway enrichment in the *Tlr4*^fl/fl^ group and the *Tlr4*^ΔIEC^ group, with or without LPS treatment. **(B)** Heatmaps of *Tlr4* and 5-HT signaling pathway-related gene set expression in the four groups. **(C)** The expression of up-regulated genes in the colon of offspring mice in the *Tlr4*^fl/fl^ and *Tlr4*^ΔIEC^ groups with or without LPS treatment during gestation was validated by quantitative PCR analysis (*n* = 5 per group; two-way ANOVA). The *Reg4* and *Sypl2* mRNA expression levels were independently affected by *Tlr4*-specific knockout or LPS in the combined *Tlr4*^fl/fl^ and *Tlr4*^ΔIEC^ groups, as well as in the combined PBS and LPS groups (*n* = 10). Data were presented as mean ± standard error of the mean. The term “Interaction” denotes the effect of LPS in the *Tlr4*^ΔIEC^ mice compared with *Tlr4*^fl/fl^ mice. ns, not significant; ∗*P* < 0.05, ∗∗*P* < 0.01, ∗∗∗*P* < 0.001, and ∗∗∗∗*P* < 0.0001. LPS, lipopolysaccharide; PBS, phosphate buffer saline; TLR4, toll-like receptor 4; 5-HT, 5-hydroxytryptamine; Reg4, regenerating family member 4; Sypl2, synaptophysin like 2.

Having found intestinal motility abnormalities and the activation of the TLR4 signaling pathway in offspring mice exposed to LPS during pregnancy, we performed RNA sequencing analysis to identify altered pathways and DEGs in colon tissue. RNA sequencing analysis revealed a total of 824 DEGs, including 506 up-regulated and 318 down-regulated genes, between the LPS and PBS groups in *Tlr4*^fl/fl^ offspring mice, and 683 DEGs, including 406 up-regulated genes and 277 down-regulated genes in the *Tlr4*^ΔIEC^ offspring mice (Fig. 4A). In addition, KEGG pathway enrichment analysis of DEGs in the *Tlr4*^fl/fl^ offspring mice revealed the enrichment of four relevant pathways: serotonergic synapse, NF-κB signaling pathway, Toll-like receptor signaling pathway, and tryptophan metabolism (Fig. 4A). The expression of DEGs involved in these four pathways was higher in the prenatal LPS-exposed *Tlr4*^fl/fl^ offspring mice than in the prenatal PBS-exposed *Tlr4*^fl/fl^ offspring mice. We also identified Tlr4-and 5-HT-related gene sets and performed gene cluster expression analysis in tissue samples of the four groups. A high level of expression of TLR4-and 5-HT-related genes was detected in the intestinal tract of prenatal LPS-exposed *Tlr4*^fl/fl^ offspring mice, but not in the colon of prenatal LPS-exposed *Tlr4*^ΔIEC^ offspring mice (Fig. 4B). The RNA sequencing results were validated by measuring mRNA levels of a few selected genes in the four groups by quantitative PCR analysis (Fig. 4C). The expression level of *Tlr4* mRNA in the colon of prenatal LPS-exposed *Tlr4*^fl/fl^ offspring mice was significantly higher than that in the PBS group (*P* = 0.0194), and there was no significant difference between the two *Tlr4*^ΔIEC^ groups (with or without LPS exposure) during pregnancy. This finding aligns with our observation of the activation of the TLR4 pathway. The 5-hydroxytryptamine receptor 4 (*Htr4*) is a key molecule involved in the regulation of intestinal motility.^31^ The intestinal mRNA expression level of *Htr4* was significantly higher in *Tlr4*^fl/fl^ offspring mice of the LPS group than that in those of the PBS group (*P* = 0.0074). However, the intestinal mRNA expression level of *Htr4* could not be increased by prenatal LPS exposure after intestinal-specific knockout of *Tlr4* (Fig. 4C). Furthermore, Bonferroni post hoc analysis revealed a significant effect of the interaction between LPS exposure and *Tlr4* knockout on the *Tlr4* and *Htr4* mRNA expression levels (*P* = 0.0166 and *P* = 0.0005, respectively). Regenerating family member 4 (*Reg4*)^32^ and synaptophysin-like 2 (*Sypl2*) are two new biomarkers for EC cells. Subsequent examination of the mRNA expression levels of *Reg4* and *Sypl2* revealed that their mRNA expression levels in the colon were significantly increased in LPS-exposed *Tlr4*^fl/fl^ offspring mice (*P* = 0.0317 and *P* = 0.0096, respectively), but LPS exposure did not lead to these significant changes after *Tlr4*-specific knockout (Fig. 4C). After combining the PBS and LPS groups or the *Tlr4*^ΔIEC^ and *Tlr4*^fl/fl^ groups, the mRNA expression level of *Reg4* was significantly increased in the combined LPS group compared with that in the combined PBS group (*P* = 0.0116), suggesting that the main effect of LPS on the *Reg4* mRNA expression level is independent of *Tlr4* knockout. Additionally, the expression level of *Sypl2* mRNA in the combined *Tlr4*^ΔIEC^ group was significantly decreased compared with in the combined *Tlr4*^fl/fl^ group (*P* = 0.0001), indicating that the main effect of *Tlr4* knockout on the *Sypl2* mRNA expression level is independent of LPS exposure.

Prenatal LPS-exposed *Tlr4*^fl/fl^ offspring mice had activated 5-HT pathways in the colon, which were not activated in prenatal LPS-exposed *Tlr4*^ΔIEC^ offspring mice.

### Prenatal exposure to LPS induced activation of the 5-HT signaling pathway in the *Tlr4*^fl/fl^ offspring but had no impact on the *Tlr4*^ΔIEC^ offspring

**Figure 5:**
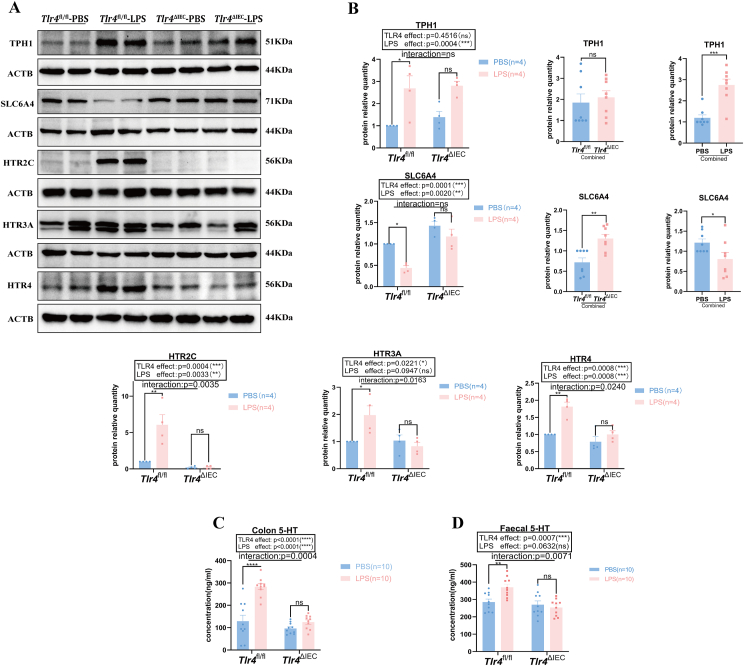
The expression levels of colonic 5-HT-related proteins in *Tlr4*^fl/fl^ and *Tlr4*^ΔIEC^ offspring mice with or without prenatal LPS exposure. **(A)** The protein expression levels of TPH1, SLC6A4, HTR2C, HTR3A, and HTR4 in the four groups. **(B)** TPH1, SLC6A4, HTR2C, HTR3A, and HTR4 protein expression levels normalized to ACTB (*n* = 4 per group; two-way ANOVA). In the combined *Tlr4*^fl/fl^ and *Tlr4*^ΔIEC^ groups, as well as in the combined PBS and LPS groups (*n* = 8), TPH1 and SLC6A4 expression levels were independently affected by *Tlr4*-specific knockout or LPS exposure. **(C, D)** With or without prenatal LPS exposure, 5-HT concentrations in colon and fecal samples of *Tlr4*^fl/fl^ and *Tlr4*^ΔIEC^ offspring mice were detected by ELISA (*n* = 10 per group; two-way ANOVA). Data were presented as mean ± standard error of the mean. The term “Interaction” denotes the effect of LPS in the *Tlr4*^ΔIEC^ mice compared with *Tlr4*^fl/fl^ mice. ns, not significant; ∗*P* < 0.05, ∗∗*P* < 0.01, ∗∗∗*P* < 0.001, and ∗∗∗∗*P* < 0.0001. LPS, lipopolysaccharide; PBS, phosphate buffer saline; 5-HT, 5-hydroxytryptamine; TPH1, tryptophan hydroxylase 1; SLC6A4, solute carrier family 6 member 4; HTR2C, 5-hydroxytryptamine receptor 2C; HTR3A, 5-hydroxytryptamine receptor 3A; HTR4, 5-hydroxytryptamine receptor 4; ACTB, actin beta.

To evaluate the changes in 5-HT levels and signaling pathways in the intestinal tract of prenatal LPS-exposed offspring mice, we first analyzed the protein levels of the rate-limiting enzyme tryptophan hydroxylase (TPH1). As shown in Figure 5A, exposure to LPS led to a significant up-regulation of TPH1 protein expression in *Tlr4*^fl/fl^ mice (*P* = 0.0192), but no statistically significant difference in TPH1 protein expression was found in the *Tlr4*^*ΔIEC*^ offspring, although an uptrend in the TPH1 protein expression level was observed after LPS exposure. After combining the *Tlr4*^fl/fl^ and *Tlr4*^ΔIEC^ groups, the TPH1 protein expression level was significantly increased in the combined LPS group compared with that in the combined PBS group (*P* = 0.0002) (Fig. 5A, B), suggesting that the main effect of LPS on the TPH1 protein expression level is independent of *Tlr4* knockout. In addition, the protein expression levels of SLC6A4 were significantly decreased in the *Tlr4*^fl/fl^ offspring mice after LPS exposure (*P* = 0.0139), and there was no statistically significant difference between the LPS and PBS exposures of the mice in the *Tlr4*^ΔIEC^ group. After combining the PBS and LPS groups or the *Tlr4*^ΔIEC^ and *Tlr4*^fl/fl^ groups, the expression level of SLC6A4 in the combined *Tlr4*^ΔIEC^ group was significantly increased compared with that in the combined *Tlr4*^fl/fl^ group (*P* = 0.0017), while in the combined LPS group, the expression of SLC6A4 was significantly decreased compared with that in the combined PBS group (*P* = 0.0474) (Fig. 5A, B).

Furthermore, prenatal LPS-exposed *Tlr4*^fl/fl^ mice expressed high levels of 5-hydroxytryptamine receptor 2C (HTR2C), 5-hydroxytryptamine receptor 3A (HTR3A), and 5-hydroxytryptamine receptor 4 (HTR4) in the intestinal tract (*P* = 0.0014, *P* = 0.0412, and *P* = 0.0019, respectively), but no changes in the intestinal expression of these receptors were observed in *Tlr4*^ΔIEC^ offspring mice with or without LPS exposure (Fig. 5A, B). Furthermore, the Bonferroni post hoc analysis revealed a significant effect of the interaction between the LPS treatment and *Tlr4*^ΔIEC^ mice on the expression of these 5-HT receptors (*P* = 0.0035, *P* = 0.0163, and *P* = 0.0240, respectively). Subsequent measurement of the levels of 5-HT in colon tissue and feces revealed that prenatal LPS-exposed *Tlr4*^fl/fl^ offspring mice showed a remarkable increase in 5-HT levels in colon tissue and feces (*P* < 0.0001 and *P* = 0.0092, respectively), whereas *Tlr4*^ΔIEC^ offspring mice, regardless of LPS exposure, showed no significant increase in 5-HT levels (Fig. 5C, D). Additionally, there was a significant effect of the interaction between LPS exposure and *Tlr4*-specific knockout on the levels of 5-HT both in colon and fecal samples (*P* = 0.0004 and *P* = 0.0071, respectively).

Prenatal LPS exposure activated the 5-HT signaling pathway in the intestines of *Tlr4*^fl/fl^ offspring mice, increasing the level of the enzyme that limits 5-HT synthesis and decreasing the level of the transporter protein. LPS treatment and *Tlr4* knockout influenced the expression of the 5-HT receptor and intestinal levels of 5-HT, but the *Tlr4*^ΔIEC^ group showed no significant changes in 5-HT pathway activation.

### TLR4 and 5-HT levels were higher in the LPS-exposed BON-1 cells, but these LPS-induced effects were abolished by TAK-242 treatment

**Figure 6:**
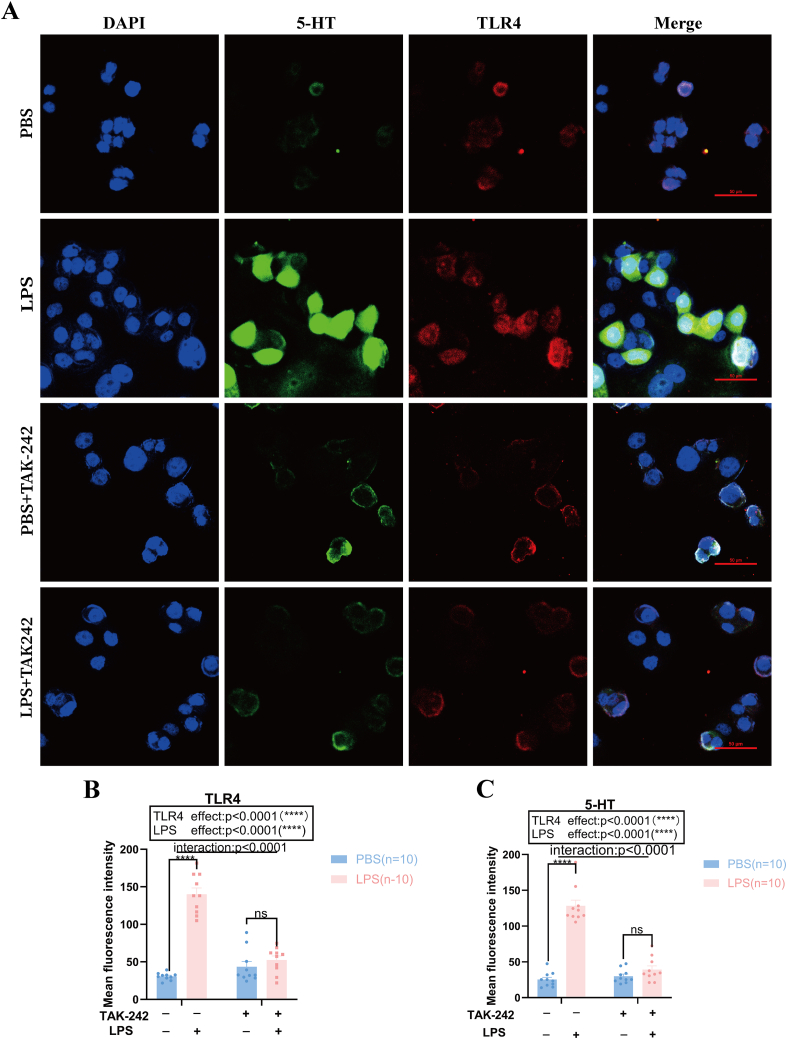
Levels of 5-HT and TLR4 in BON-1 cells treated with LPS or PBS combined with TAK-242. **(A)** Confocal microscopy imaging showing that BON-1 cells treated with PBS and LPS with or without TAK-242 (a TLR4 inhibitor) co-localized with 5-HT (green) and TLR4 (red). Scale bar = 50 μm (*n* = 10 per group; 400 × ). **(B, C)** Quantification of the mean fluorescence intensity for TLR4 and 5-HT (*n* = 10 per group). Data are expressed as mean ± standard error of the mean; ns, not significant; ∗∗∗∗*P* < 0.0001. 5-HT, 5-hydroxytryptamine; TLR4, toll-like receptor 4; LPS, lipopolysaccharide; PBS, phosphate buffer saline.

BON-1 cells were used to confirm the co-localization and changes in the levels of TLR4 and 5-HT by immunofluorescence staining. As shown in Figure 6A, LPS treatment significantly increased the levels of 5-HT and TLR4 in BON-1 cells. Additionally, the treatment with the TLR4 inhibitor TAK-242 effectively mitigated this LPS-induced effect. We quantified the fluorescence intensities of TLR4 and 5-HT, as shown in Figure 6B. LPS increased the mean fluorescence intensity of TLR4 and 5-HT in BON-1 cells compared with PBS (*P* < 0.0001 and *P* < 0.0001, respectively), while TAK-242 blocked this effect. The interaction between LPS treatment and TAK-242 treatment had a significant effect on the fluorescence intensity of TLR4 and 5-HT (*P* < 0.0001 and *P* < 0.0001, respectively).

These results suggest that LPS stimulation can increase the levels of TLR4 and 5-HT in BON-1 cells, and the TLR4 inhibitor TAK242 can block this effect.

### The interaction between TLR4 and PIEZO1 proteins facilitated calcium influx in LPS-treated BON-1 cells, potentially leading to the secretion of 5-HT

**Figure 7:**
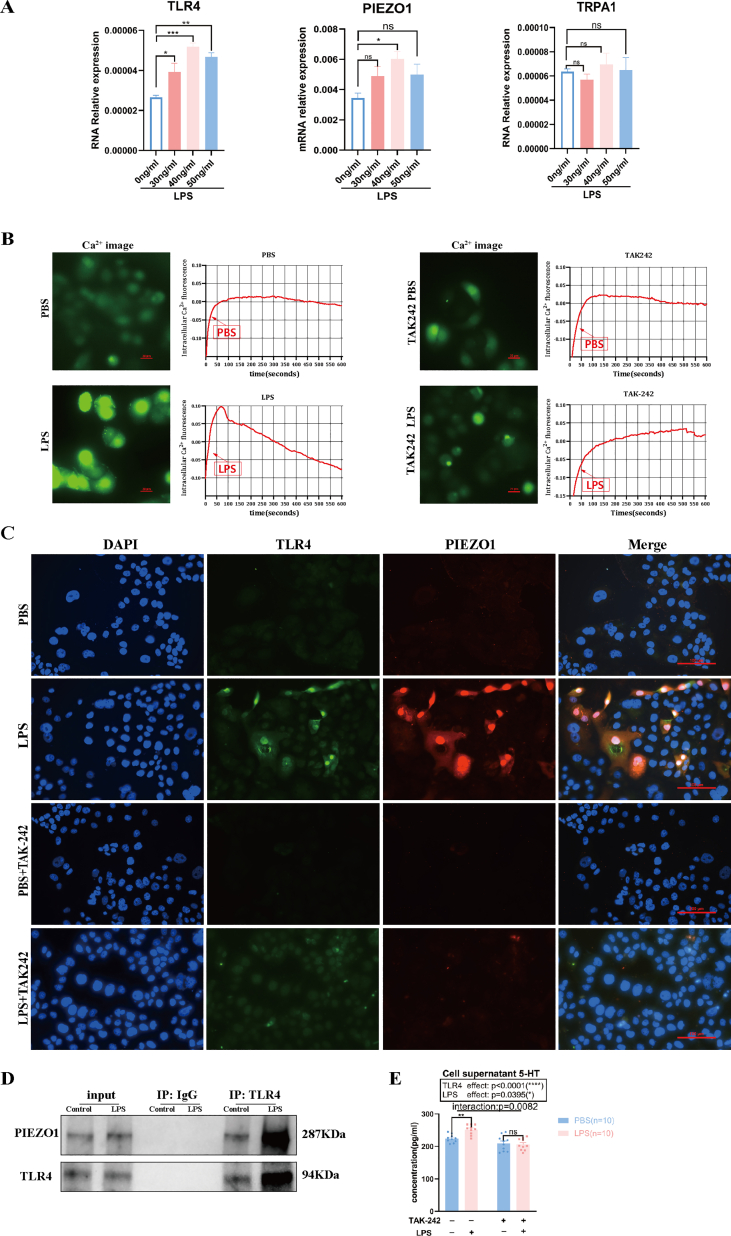
TLR4 binds with PIEZO1 to promote calcium influx in BON-1 cells. **(A)** The mRNA expression levels of *TLR4*, *PIEZO*1, and *TRPA1* in BON-1 cells treated with different concentrations of LPS were determined by quantitative PCR analysis (*n* = 5 per group; one-way ANOVA). **(B)** Calcium images showing calcium influx changes in the BON-1 cells treated with PBS or LPS combined with or without the TLR4 inhibitor TAK-242. Scale bar = 50 μm (*n* = 3 per group). **(C)** The co-localization observation of TLR4 (green) and PIEZO1 (red) in the four different treatment groups of BON-1 cell by confocal microscopy. Scale bar = 100 μm (*n* = 10 per group). **(D)** TLR4 bound to PIEZO1 in the BON-1 cell treated with or without LPS as determined by co-immunoprecipitation using TLR4 or PIEZO1 antibodies. **(E)**The concentration of 5-HT in the supernatant of BON-1 cells treated with LPS or PBS, with or without treatment with TAK-242 (*n* = 10), was determined by ELISA. Data were expressed as mean ± standard error of the mean; ns, not significant; ∗*P* < 0.05, ∗∗*P* < 0.01, ∗∗∗*P* < 0.001, and ∗∗∗∗*P* < 0.0001. TLR4, toll-like receptor 4; PIEZO1, Piezo-type mechanosensitive ion channel component 1; TRPA1, transient receptor potential cation channel subfamily A member 1; LPS, lipopolysaccharide; PBS, phosphate buffer saline; 5-HT, 5-hydroxytryptamine.

We investigated the mechanism of action of TLR4 and the synthesis of 5-HT *in vitro* in BON-1 cells treated with LPS and TAK-242. Initially, quantitative PCR analysis was performed to determine the mRNA expression levels of *Tlr4* and proteins associated with calcium channels in BON-1 cells following LPS stimulation. The results, shown in Figure 7A, revealed that the mRNA expression levels of both *TLR4* and *PIEZO1* were significantly induced by LPS at 40 ng/mL in BON-1 cells (*P* = 0.0004 and *P* = 0.0273, respectively). However, no significant statistical difference was found in the mRNA expression levels of the transient receptor potential cation channel subfamily A member 1 (*TRPA1)* after treatment with different concentrations of LPS. Calcium imaging revealed that LPS stimulation induced marked calcium influx in BON-1 cells, while treatment with TAK-242 attenuated the LPS-induced calcium influx (Fig. 7B). We also evaluated the relationship between the protein expression of TLR4 and the ion channel protein PIEZO1 in BON-1 cells after LPS exposure. Immunofluorescence staining revealed increased co-localization of TLR4 and PIEZO1 proteins in BON-1 cells treated with LPS. However, treatment with TAK-242 resulted in decreased co-expression of TLR4 and PIEZO1 proteins in response to LPS (Fig. 7C). Additionally, co-immunoprecipitation analysis of the protein–protein interaction between TLR4 and PIEZO1, using the anti-TLR4 antibody to precipitate the PIEZO1, revealed that PIEZO1 was strongly bound to TLR4 in LPS-treated BON-1 cells (Fig. 7D). We also measured the concentration of 5-HT in the cell supernatant and found that it was increased by LPS treatment compared with PBS treatment (*P* = 0.0078), but this effect was blocked by TAK242, revealing a significant effect of the interaction between LPS and TAK-242 treatments on the 5-HT levels in the cell supernatant (*P* = 0.0082) (Fig. 7E).

According to these findings, LPS stimulation up-regulated the expression levels of TLR4 and PIEZO1 and facilitated their interaction in BON-1 cells, triggering cellular calcium influx and possibly leading to the release of 5-HT.

### PIEZO1 was highly expressed in the colon of *Tlr4*^fl/fl^ offspring mice exposed to LPS during pregnancy but not in *Tlr4*^ΔIEC^ mice

**Figure 8:**
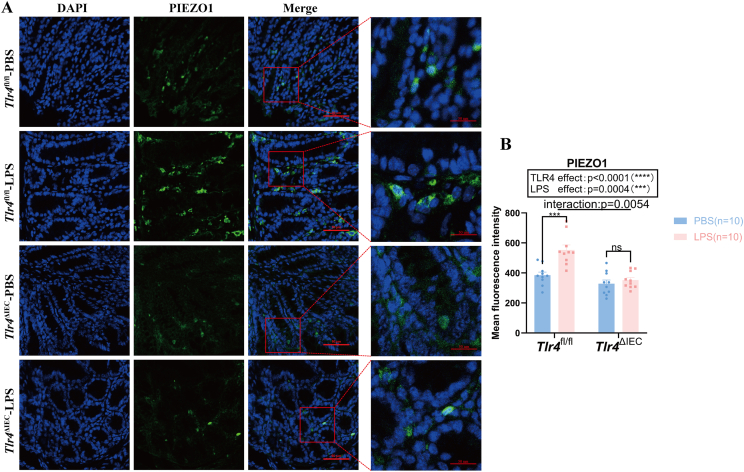
The expression of PIEZO1 in the colonic mucosa of *Tlr4*^fl/fl^ and *Tlr4*^ΔIEC^ offspring mice with or without prenatal LPS exposure. **(A)** Representative confocal immunofluorescence microscopy images for PIEZO1 (green) in the colonic mucosa of prenatal LPS- or PBS-exposed offspring mice in the *Tlr4*^fl/fl^ and *Tlr4*^ΔIEC^ groups. Scale bar = 50 μm (*n* = 10 per group). **(B)** Quantification of the mean fluorescence intensity for PIEZO1 (*n* = 10 per group). Data were expressed as mean ± standard error of the mean; ns, not significant; ∗∗∗*P* < 0.001 and ∗∗∗∗*P* < 0.0001. PIEZO1, Piezo-type mechanosensitive ion channel component 1; LPS, lipopolysaccharide; PBS, phosphate buffer saline.

The expression of PIEZO1 in the colonic mucosa of offspring mice was detected by immunofluorescence staining. As shown in Figure 8A, the expression of PIEZO1 in the colonic mucosa of offspring mice exposed to LPS during pregnancy in the *Tlr4*^fl/fl^ group was significantly higher than in the PBS group (*P* = 0.0001). In addition, there was no difference in the expression of PIEZO1 between the LPS and PBS groups in *Tlr4*^ΔIEC^ mice. The mean values of the quantification of the immunofluorescence intensity of PIEZO1 are shown in Figure 8B. There was an interaction between LPS exposure and *Tlr4* knockout in the intestinal epithelium (*P* = 0.0054).

These results suggest that LPS exposure during pregnancy increased the expression of PIEZO1 in the colonic mucosa of offspring mice. However, *Tlr4* knockout in the intestinal epithelium did not have this effect.

## Discussion

This study found increased fecal water content and accelerated intestinal motility in prenatal LPS-exposed offspring mice. However, prenatal LPS exposure did not improve the intestinal motility of offspring mice after the conditional knockout of intestinal epithelial *Tlr4*. These results suggested that intestinal epithelial TLR4 is involved in intestinal motility abnormalities in prenatal LPS-exposed offspring mice.

A healthy maternal prenatal environment plays a crucial role in the healthy development of offspring.^1^, ^2^, ^3^ Numerous studies have reported that prenatal infection is associated with abnormal intestinal function in offspring. It was found that prenatal inflammation caused by prenatal exposure to LPS could damage the integrity of the intestinal barrier in offspring, which persists into adulthood.^9^ Another study found that infections during pregnancy increased the susceptibility of offspring to intestinal inflammatory diseases.^7^ Although several studies have observed changes in the offspring gut, few have specifically focused on offspring gut motility. Normal intestinal motility, essential for nutrient absorption and waste expulsion, facilitates digestive tract propulsion of ingested material.^10^ Considering the lack of research on offspring gut motility, we sought to conduct this study.

While gut epithelial TLR4 is primarily responsible for maintaining intestinal immune barrier homeostasis, its influence on intestinal motility remains underexplored. The diarrhea-predominant irritable bowel syndrome (IBS-D) model rats showed accelerated intestinal motility accompanied by an increase in TLR4 expression.^33^ Other studies have found that TLR4 is involved in the neurotransmitter-induced contractile response, including acetylcholine and opioid-induced intestinal peristalsis, and the corresponding peristaltic response was reduced after *Tlr4* knockout.^34^^,^^35^ These studies suggest that TLR4 participates in the regulation of intestinal motility. LPS naturally activates TLR4. Research showed that LPS significantly reduced the longitudinal smooth muscle contractions of the duodenum induced by acetylcholine in rabbits, but this effect could be reduced by pyrrolidinedithiocarbamate (PDTC), a RELA inhibitor.^36^ Conversely, other studies have reported increased colonic contractions in rats with systemic inflammation,^37^ making the link between inflammation and intestinal motility unclear. We also investigated the precise mechanism by which TLR4 regulates intestinal dynamics in offspring mice exposed to prenatal infection. After prenatal exposure to LPS, the expression of TLR4 and its downstream proteins in the colon of offspring mice increased, but no corresponding changes were observed in the *Tlr4*^ΔIEC^ group. Colon tissues from offspring mice were analyzed by RNA sequencing. The results revealed elevated TLR4 and 5-HT signaling levels in prenatal LPS-exposed offspring mice compared with prenatal PBS-exposed offspring mice in the *Tlr4*^fl/fl^ group. In contrast, prenatal LPS exposure did not result in increased TLR4 and 5-HT signaling levels in the colon of offspring mice in the *Tlr4*^ΔIEC^ group. These findings suggest that TLR4 and 5-HT signaling are connected. We hypothesized that in LPS-exposed offspring mice, the gastrointestinal tract may accelerate peristalsis due to enhanced activation of 5-HT signaling pathways.

The neurotransmitter 5-HT is an important regulator of various physiological processes, including mood regulation, sleep cycles, appetite control, learning, and memory, and its role in promoting intestinal motility has been widely recognised.^38^ It plays a role in promoting intestinal motility mainly by binding to its corresponding receptors expressed in the intestine.^19^^,^^20^ Therefore, many drugs targeting 5-HT receptors expressed in the gut have been developed for treating diseases related to intestinal motility abnormalities.^31^ In our subsequent experiments, we verified that the expression levels of 5-HT receptors and the content of 5-HT in colon tissues and fecal samples of prenatal LPS-exposed offspring mice of the *Tlr4*^fl/fl^ group were increased compared with the prenatal PBS-exposed offspring mice, which may be the main reason for the acceleration of intestinal motility. However, in the *Tlr4*^ΔIEC^ group, there was no corresponding change. High prenatal infection-induced expression of TLR4 has been reported in different tissues of offspring.^39^ Following maternal LPS exposure, we also observed high intestinal expression of TLR4 and its downstream proteins. However, there is no precise mechanism that explains how TLR4 regulates the elevated levels of 5-HT in the colon of prenatal LPS-exposed offspring mice.

In the human body, 95% of 5-HT comes from the intestine, mainly secreted by EC cells.^12^ EC cells produce 5-HT to enhance signal transmission between the gut and brain.^40^ In this study, we focused on intestinal EC cells to investigate the specific mechanism by which TLR4 regulates the activation of the intestinal 5-HT signaling pathway in offspring mice. Previous studies indicate that while EC cells primarily secrete 5-HT dependent on calcium influx, some cells also release it independent of calcium influx.^41^ It has been demonstrated that IL-33 promotes phospholipase C gamma 1 (PLC-γ1) to increase intracellular calcium ions and affect the secretion of 5-HT in EC cells.^17^ Accordingly, we hypothesized that TLR4 might affect the secretion of 5-HT by EC cells by regulating calcium influx. BON-1 is widely used as a model cell for EC cells.^42^^,^^43^ In the present study, we used the BON-1 cell line to investigate the possible mechanism by which TLR4 regulates 5-HT secretion. Our study revealed that LPS-treated BON-1 cells expressed higher levels of TLR4 and PIEZO1, and TAK-242 inhibited these LPS-induced responses. The mechanosensitive PIEZO1 is a member of a distinct nonselective cationic mechanosensitive channel protein family expressed in mammalian cells,^44^ which regulates various physiological processes.^45^ The PIEZO1 cation channel regulates calcium influx and drives various vesicle release processes, including cytokine release from astrocytes^46^ and growth factor secretion from macrophages.^47^ A recent study found that the cation channel PIEZO1 acts as a sensor of single-stranded RNA, regulating 5-HT production in the gut.^48^ Also, recent research has revealed that the PIEZO1 protein forms a complex with TLR4 on the surface of macrophages, effectively inhibiting downstream signaling from TLR4 and dampening macrophage activation.^49^ Additionally, another study suggested that in adipose cells, PIEZO1 exhibits anti-inflammatory properties that further prevent TLR4 activation.^50^ However, there is a lack of reported research on whether TLR4 regulates PIEZO1 through post-transcriptional regulation by activating downstream signaling pathways. We suspect that there is a direct interaction between the cell membrane proteins TLR4 and PIEZO1. Our study found that LPS stimulation increased TLR4 expression, and the TLR4-PIEZO1 binding enhanced calcium influx in BON-1 cells, leading to increased 5-HT secretion. Additionally, LPS exposure during pregnancy increased PIEZO1 expression in the colonic mucosa of offspring mice, but this effect was absent in intestinal epithelial-specific *Tlr4* knockout mice.

We found that TLR4 regulates intestinal motility by increasing calcium influx in EC cells after binding to PIEZO1 channels and then promoting 5-HT secretion. This may explain the effect of TLR4 on intestinal motility in prenatal LPS-exposed offspring mice. However, we did not explore calcium influx-independent 5-HT secretion, and the lack of female offspring is a study limitation.^51^ The effects of TAK-242 on calcium influx *in vivo* are unclear and need more research, and the mechanism of intestinal TLR4 in regulating 5-HT signaling is also unknown. Our research will continue to focus on suppressing PIEZO1 *in vivo*, which is our next research direction.

In summary, we found an acceleration of intestinal motility in prenatally infected offspring mice, and the TLR4 protein might contribute to high intestinal motility by promoting 5-HT secretion by binding to PIEZO1. Therefore, targeting TLR4 in the intestinal epithelium may alleviate the state of intestinal motility abnormalities in prenatally infected offspring.

## CRediT authorship contribution statement

**Ruifang Luo:** Writing – original draft, Methodology, Investigation, Formal analysis, Data curation. **Yuan Miao:** Supervision, Data curation. **Riqiang Hu:** Supervision, Methodology, Investigation. **Fang Lin:** Supervision, Methodology. **Junyan Yan:** Visualization, Methodology, Formal analysis. **Ting Yang:** Supervision, Software. **Lu Xiao:** Supervision, Methodology. **Zhujun Sun:** Supervision. **Yuting Wang:** Project administration. **Jie Chen:** Writing – review & editing, Supervision, Project administration, Funding acquisition, Conceptualization.

## Funding

This study was supported by the 10.13039/501100001809National Natural Science Foundation of China (No. 82372559, 31971089) and the General Project of Chongqing Natural Science Foundation (China) (No. CSTB2022NSCQ-MSX0107).

## Conflict of interests

The authors declared no competing interests.

## Figures and Tables

**Table S1:**
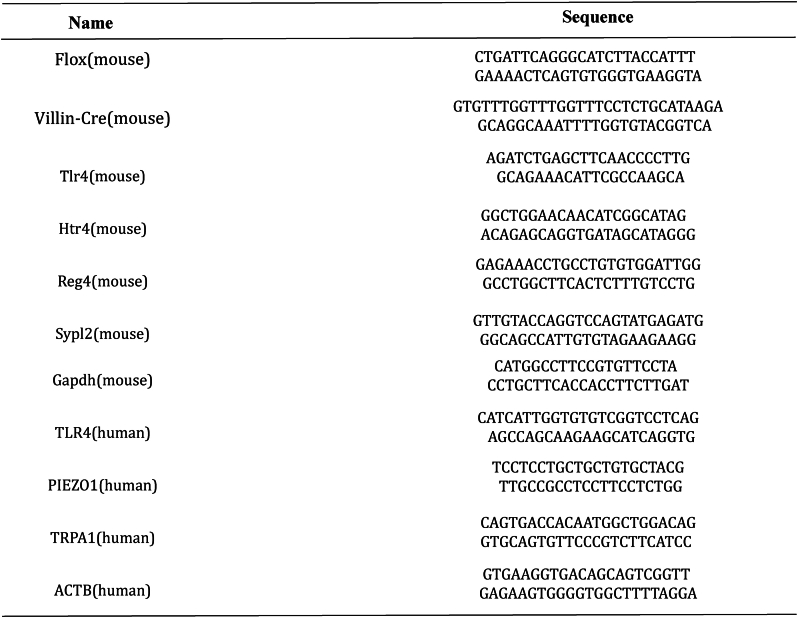
The primers used in this study.
